# An observation of a negative effect of social cohesion on creativity in musical improvisation

**DOI:** 10.1038/s41598-024-52350-7

**Published:** 2024-02-05

**Authors:** Adrian Kempf, Mathias Benedek, Andrea Schiavio

**Affiliations:** 1 Department of Psychology, University of Graz, Glacisstraße 27, 8010 Graz, Austria; 2 Department of Psychology, University of Graz, Graz, Austria; 3 School of Arts and Creative Technologies, University of York, York, UK

**Keywords:** Human behaviour, Cooperation

## Abstract

Although various social factors can significantly impact creative performance, it is still unclear how social cohesion (i.e., how close we feel to others) influences creativity. We therefore conducted two studies exploring the association between social cohesion and creativity within the domain of musical improvisation, a prime example of creative performance, which usually plays out in social contexts. The first study (n = 58 musical novices) showed that music-induced synchrony facilitates social cohesion. In our second study (n = 18 musical novices), we found that in two out of three experimental conditions, increased social cohesion is associated with less creative musical outcomes, as rated by nine expert musicians. In our subsequent analysis we related measures of social cohesion and creativity. This approach highlights how, within a musical setting, creativity unfolds in the context of social contingencies as social cohesion and related factors.

## Introduction

Human creativity has been traditionally defined as a phenomenon that gives rise to processes and outcomes that are novel and appropriate^1^. Among others, recent empirical and theoretical work in organizational psychology (e.g., Hennessey and colleagues^2^), educational science^3,4^, as well as creativity research^5^, strongly emphasizes the key role which social contingencies play in creative thought and action. This scholarship asks us to consider instances where creativity is inherently determined by social factors; for example, cases in which we compare our own ideas with those put forward by others, and occasions where creative activity (e.g., dancing) is evaluated in light of the social environment in which such an activity takes place^6,7^. With this in mind, a particularly fascinating topic emerging from this line of research is that of social cohesion: how do closeness and social proximity between people shape creativity? On the one hand, social cohesion has been shown to positively correlate with creative performance in collective tasks, as outlined in work testing convergent and divergent creative thinking^8–10^; on the other hand, it has been argued that high levels of social cohesion might lead to conformity, which may eventually inhibit creativity^11^. As such, the role of social cohesion in creativity remains unclear.

In the present contribution, we aim to address this topic via two behavioral studies which explore the influence of social cohesion on creativity in a musical context. We chose the latter setting as music is widely conceived of as an intrinsically social and creative phenomenon^12,13^, which may have evolved phylogenetically and ontogenetically for the purpose of creating social bonds, connectedness, and trust within groups (see Savage and colleagues^14^ and Trevarthen^15^), amongst other functions.

We operationalized the link between creativity and social cohesion in music by focusing on two core dimensions that lie at the heart of much musical activity: *improvisation* and *interpersonal synchronization.* The former has been often described as the epitome of artistic creativity and has been examined in a number of studies dedicated to exploring a range of creative phenomena in individuals and groups (e.g., Borgo^16^; Berkowitz and Ansari^17^; Beaty and colleagues^18^). The latter has been shown to play a key role in influencing social cohesion (see Rennung and Göritz^19^ for a review). Synchronous movement to music is known to elicit a feeling of closeness amongst group members^20,21^ as well as facilitate the ability of perspective-taking, i.e., mentally stepping into someone else’s shoes to comprehend their perspective, beliefs, or emotions^22^. Similar results have been found for interpersonal synchronization to music with virtual partners^23–25^. Intriguingly, previous research already suggested that interpersonal synchronization might also play an important role in creative group tasks such as musical improvisation^26,27^ or brainstorming^28^. Yet, the relationship between social cohesion, interpersonal synchronization, and creativity remains to be explored.

We carried out two studies to investigate the interplay between social cohesion and creativity in musical improvisation. In our first study, the main question was:

### RQ_1_

Does music-induced interpersonal synchrony with a virtual partner (a stick-figure avatar) increase social cohesion?

Based on previous research by Stupacher and colleagues^23^ and Tarr and colleagues^25^, we expected interpersonal synchrony with a virtual partner to facilitate social cohesion. We used an avatar instead of a real human partner to minimize the effect of social preferences and expectations which could have emerged when assigning a random human partner.

In our second study, we asked:

### RQ_2_

How does social cohesion, manipulated through music-induced interpersonal synchrony, affect creativity in a rhythmical improvisation task?

In three conditions, participants carried out one of three synchronization tasks either with or without the virtual partner before performing three short rhythmical improvisations. We hypothesized that musical improvisations would be significantly more creative when participants synchronized with the avatar compared to the two baseline conditions where they did not synchronize. Moreover, we expected that ratings of creativity for the improvisations would be positively correlated with the perceived closeness to the virtual partner.

## Study 1: Facilitating closeness

### Methods

#### Participants

Seventy-three participants were initially recruited via the online platform Prolific (Prolific, www.prolific.co). Inclusion criteria were: (1) Not playing a musical instrument; (2) Not having studied music; and (3) Not having practiced a musical instrument in the last 5 years. Five participants were excluded as they did not perform the tasks as instructed and ten further participants did not fit the inclusion criteria. This reduced the final sample to fifty-eight participants (14 men, 43 women, 1 preferred to give no answer; age: *M* = 38.02 years, *SD* = 13.16). All participants gave informed consent and received financial reward for their involvement in the study. The study and all procedures were approved by the ethics committee associated with research of the University of Graz and were in accordance with the statements of the Declaration of Helsinki.

We chose musical novices to avoid variance due to expertise. The initial sample size was chosen based on a power simulation. We simulated datasets in light of the findings by Stupacher and colleagues^23^ assuming that the data followed a normal distribution. The effect of interest in our power analysis was the difference between watching the virtual partner and synchronizing with them as estimated by the regression coefficient β_2_ in our (Model 1) summarized in Table 1. In the power simulation we increased the sample size in steps of 36 (the number of participants needed for a fully counterbalanced experiment) maintaining the same effect size until achieving a power level greater than 80% at a significance level of 0.05. At each step, the simulation was run 1000 times. Each time, we estimated the linear mixed model we would employ in the data analysis of our study, but utilizing the simulated dataset. After reaching the planned power level at a sample size of n = 72, we proceeded the power simulation with a fixed sample size of 72 but decreased the effect size stepwise until the power dropped below 80%. Through this analysis we determined a smallest average effect of interest^29^ for the relevant regression coefficient of β = 0.23 at a power level greater than 80% and a significance level of 0.05. As the sample size was reduced by 15 participants, we performed a sensitivity power analysis^30^ using a simulation-based approach. Hence, we reran the original power simulation with a decreased sample size and increased the effect size stepwise until reaching a power level greater than 80% at a significance level of 0.05. This analysis showed that we were still able to detect an average effect of β > 0.26 (regression coefficient of interest) at a power level greater than 80% and a significance level of 0.05.

#### Procedure

**Figure 1:**
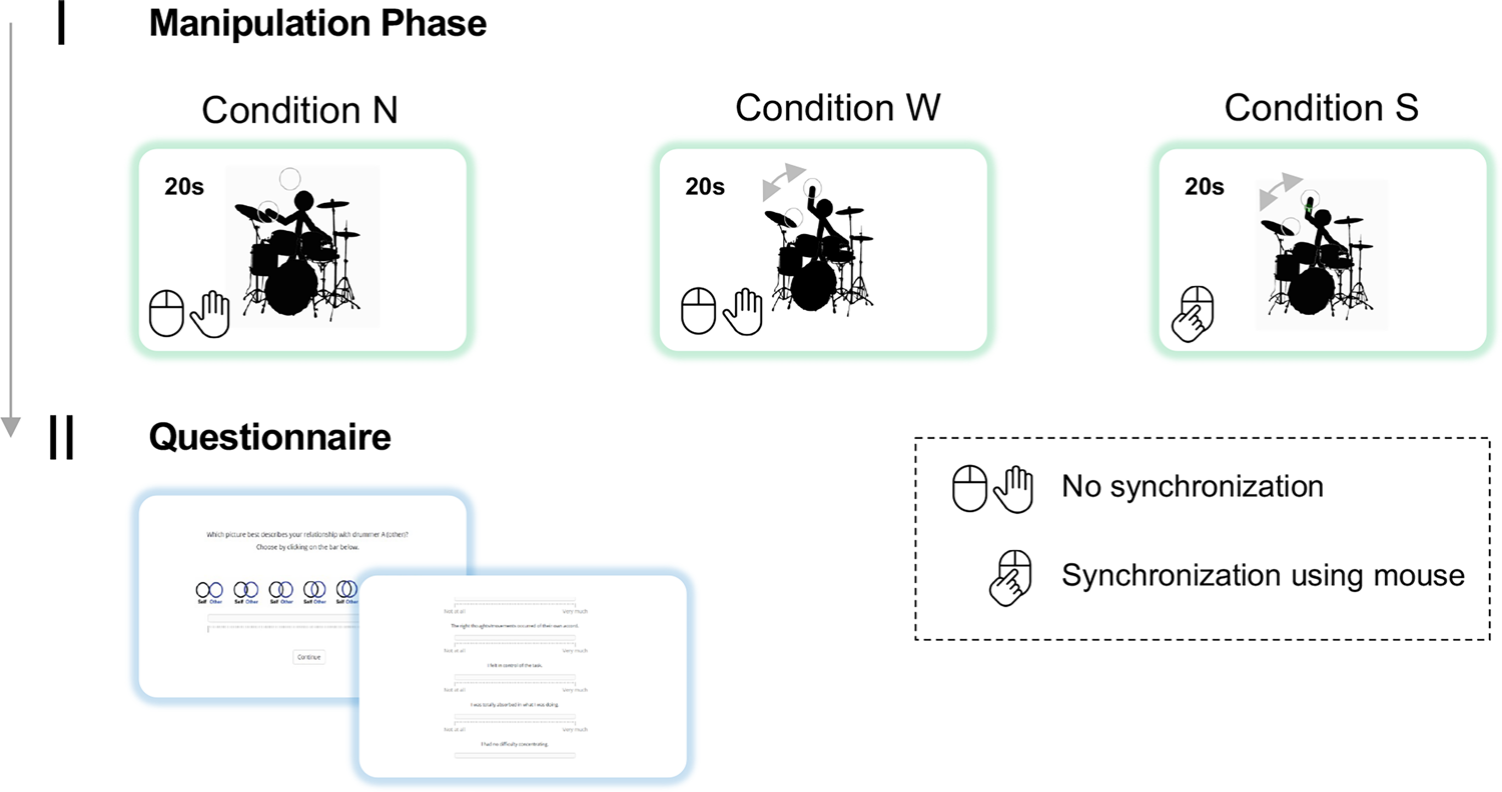
Procedure of experiment 1 involving a manipulation phase where participants interacted with a drummer avatar under different conditions followed by questionnaire phase. Participants completed all three experimental conditions including the condition N (“No-Movement”), condition W (“Watch”), and condition S (“Synchronize”). In condition “(N)o-Movement” and “(W)atch” participants did not synchronize with the drummer avatar, while in condition “(S)ynchronize” they moved their mouse cursor along with the arm of the drummer avatar. Participants ran through all conditions twice, one time in each experimental block.

The experiment consisted of two blocks in which participants engaged in three different conditions, each involving a different manipulation task followed by a questionnaire. The three conditions were repeated once per block and were fully counterbalanced across both experimental blocks (see Fig. 1). During the manipulation task of every condition, a stick figure drummer (avatar) was presented to the participant for 20 s, accompanied by an auditory stimulus (a drum track with a tempo of 120 beats per minute). Participants were invited to think of the drum beat as being actually played by the avatar. In the “*No-Movement*” condition, the avatar was standing still, and participants were instructed to sit still while listening to the drum track. In the “*Watch*” condition, the avatar was moving its arm in synchrony with the beat between two circles, one of them placed on the cymbal and one above. The drummer was hitting the cymbal on the one and three of the beat, while the arm was located in the upper circle on the two and four of the beat. Participants were again instructed to sit still while listening. In the “*Synchronize*” condition, participants moved their mouse cursor along with the drumbeat—that is, in between the two circles and in synchrony with the avatar, which was moving in a way similar to condition “*Watch*”. Participants’ movements were recorded by tracking the trajectory of their mouse cursor and visually inspected, to check for correct performance of the task.

To allow a within-subject design and avoid potential carryover effects, each avatar was given a unique color (e.g., blue), and a unique name (e.g., “Drummer A”). Participants had the chance to get accustomed to the three conditions in a test trial before the experiment. In every condition (except in the test trials), the manipulation task was followed by the same two-part questionnaire (see Supplementary Table S1). In the first part, participants were invited to rate their closeness to the avatar. Inspired by previous research showing how the effect of interpersonal synchronization on social cohesion is mediated by a self-other-overlap mechanism^31^, we conceptualized interpersonal closeness as self-other overlap, and measured it via an adapted version of the single-item “Inclusion of Other in the Self” (IOS) scale^23,32,33^. Perceived closeness was rated on a continuous slider (value range from 1 to 7 in steps of 0.1) which was placed below the IOS scale. The second part of the questionnaire included general questions on participants’ mood and on the likeability of the drummer, among others, as well as the Flow Short Scale (FSS) by Engeser and Rheinberg^34^. The FSS assesses flow in two dimensions, namely “fluency of performance” (six items) and “absorption by activity” (four items), while commonly the mean of both is used^34^.

## Results

**Figure 2:**
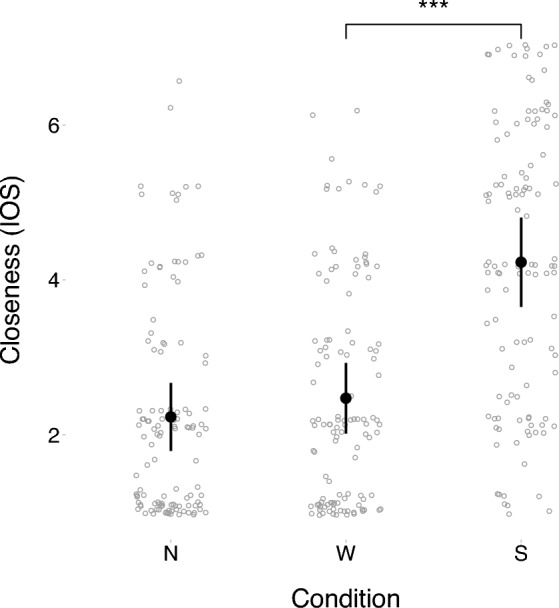
Visualization of the mean closeness (IOS) as measured in condition “(N)o-Movement”, “(W)atch” and “(S)ynchronize”. The error bars display 95% confidence intervals, corrected as proposed by Morey^65^.

To assess the effect of the manipulation task of each condition on the felt closeness to the avatar, we ran a linear mixed model using the “lmer” function from the package “lme4”^35^ (which allows fitting linear and generalized linear mixed-effect models) in R^36^. The rated closeness was used as standardized outcome variable, and experimental condition (No-movement, Watch, and Synchronize) was used as predictor. The model included a varying intercept for subject, which accounts for the variation in closeness between subjects by allowing the intercept to vary for each participant. The predictor condition was coded using reverse Helmert contrasts as it fit our hypotheses^37^. The reverse Helmert parametrization configures the linear mixed model in such a way that the intercept is the average of condition “No-Movement” and “Watch”. Furthermore, the parametrization tests for the difference between the condition “Synchronize” and the average of the conditions “No-Movement” and “Watch” as we did not expect the latter two conditions to differ in felt closeness. Moreover, the reverse Helmert coding also lets us compare the conditions “No-Movement” and “Watch” to verify our assumption that they do not differ, and control for any possible effect of simply seeing the virtual avatar move. The different levels of closeness in each condition are reported in Fig. 2. As expected, we observed significantly higher closeness (β_2_ = 1.06, 95% CI [0.92, 1.20]) for the movement condition “Synchronize” compared to the mean of the no-movement conditions “No-Movement” and “Watch”. No significant difference in felt closeness (β_1_ = 0.14, 95% CI [− 0.02, 0.29]) was found between these no-movement conditions (“No-Movement” vs. “Watch”). A detailed summary of the linear model can be found in Table 1.

**Table 1:**
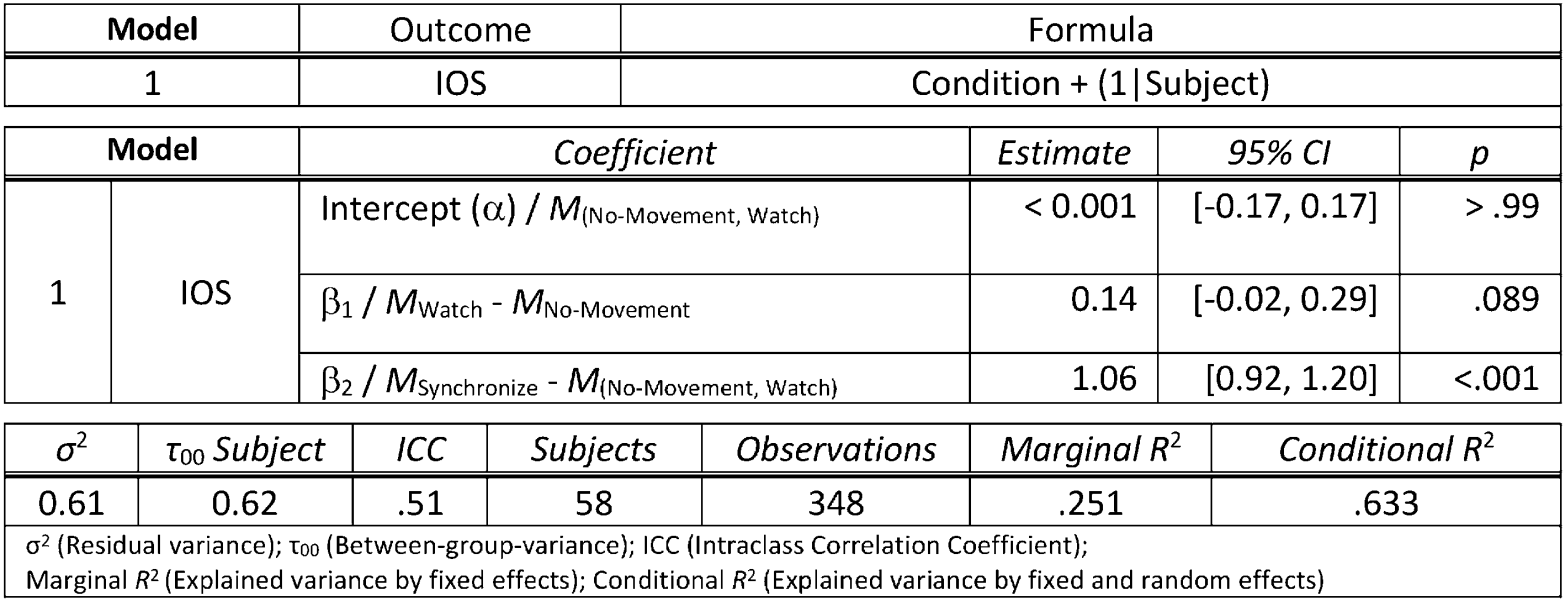
Summary of the linear mixed model (presentation similar to^66^) to assess the effect of condition on felt closeness (IOS) to the drummer. The model uses Helmert contrasts to test for: (1) the difference between the mean IOS in the no-movement conditions “(N)o-Movement” and “(W)atch” and (2) the difference between the mean IOS in movement condition “(S)ynchronize” and the mean IOS of the no-movement conditions “(N)o-Movement” and “(W)atch”. The intercept of the model is the mean IOS of the no-movement conditions. The model formula is stated in the lme4 syntax.

## Study 2: Rhythmic improvisation with avatar

### Methods

#### Participants

For the second study, we recruited a total of 30 participants: 21 musical novices were invited to perform an improvisation task (performers); nine expert musicians assessed the resulting musical improvisations (raters). Out of the 21 performers, two were excluded from the sample based on the inclusion criteria identical to study 1: (1) Not playing a musical instrument; (2) Not having studied music and (3) Not having practiced a musical instrument in the last 5 years. Another single participant was removed due to low-effort responses on the questionnaires (i.e., providing the same response on all questionnaire items within a very short period of time). The final sample of performers included eighteen participants (9 men, 9 women; age: *M* = 36.83 years, *SD* = 14.86). This sample size was chosen to allow the expert raters to assess all the improvisations in less than 90 min.

The rater group included nine musical experts (7 men, 2 women; age: *M* = 32.11 years, *SD* = 9.32). Inclusion criteria were: (1) Studying music; and (2) Playing a musical instrument for 5 or more years. The number of raters was increased from 5 to 9 to ensure an interrater reliability of at least .70 for the five most important assessment rating questions. Interrater reliability was assessed by calculating the intraclass correlation coefficients (ICC) as described by Koo and Li^38^. ICC estimates and the 95% confidence intervals were calculated with the help of the R package “psych”^39^ on base of a mean rating (k = 9), absolute agreement, two-way mixed effect model. An ICC of around .70 was chosen as the cut-off point for the inter-rater reliability as it is a typical value in studies using subjective assessment of creativity^40^. All participants gave informed consent and received financial reward for their involvement in the study. The study and all procedures were approved by the ethics committee associated with research of the University of Graz and were in accordance with the statements of the Declaration of Helsinki.

#### Tasks and Procedure

The experiment involved three fully counterbalanced conditions, each featuring a distinct manipulation task. The manipulation task of condition “Watch” involved the avatar moving its arm in synchrony with the beat between two circles, one placed on the cymbal and one above. The avatar was hitting the cymbal on the one and three of the beat, while the arm was located in the upper circle on the two and four of the beat. Participants were asked to sit still and listen to the drumbeat played by the avatar for 20 s. Condition “Dot” featured an extended manipulation task with the first part being the “Watch” task, followed by a second part where participants moved their mouse cursor synchronously to a moving dot along with a metronome click track for 20 s. The dot moved between two circles that were similarly positioned as the circles between which the avatar moves in condition “Watch”. The manipulation task of condition “Synchronize” involved the same moving avatar and backing track stimuli as in condition “Watch”; however, participants were invited to move their mouse cursor synchronously to the arm of the avatar in between the two circles along with the drumbeat for 20 s. Movement trajectory and frequency (120 beats per minute) were the same in condition “Dot” and “Synchronize”. Participants’ movements were recorded in condition “Dot” and “Synchronize” by tracking the trajectory of their mouse cursor, and visually inspected to check for correct performance of the task. Condition “Dot” was included in the experiment to control for effects caused solely by movement of the avatar, in comparison to interpersonal movement with the avatar in condition “Synchronize”. Each condition also included a common improvisation task together with the avatar. The improvisation task started with 4 s where participants could listen to the drum track followed by 15 s where they could improvise using a virtual set of two differently pitched congas. Each of the congas could be played by pressing an assigned button on the participants’ keyboard, which triggered the recording of a single open tone stroke. Participants were invited to be creative and freely improvise rhythmic patterns over the drum backing track by playing the two virtual congas. During the whole improvisation trial, participants could see the avatar moving along to the drum backing track.

**Figure 3:**
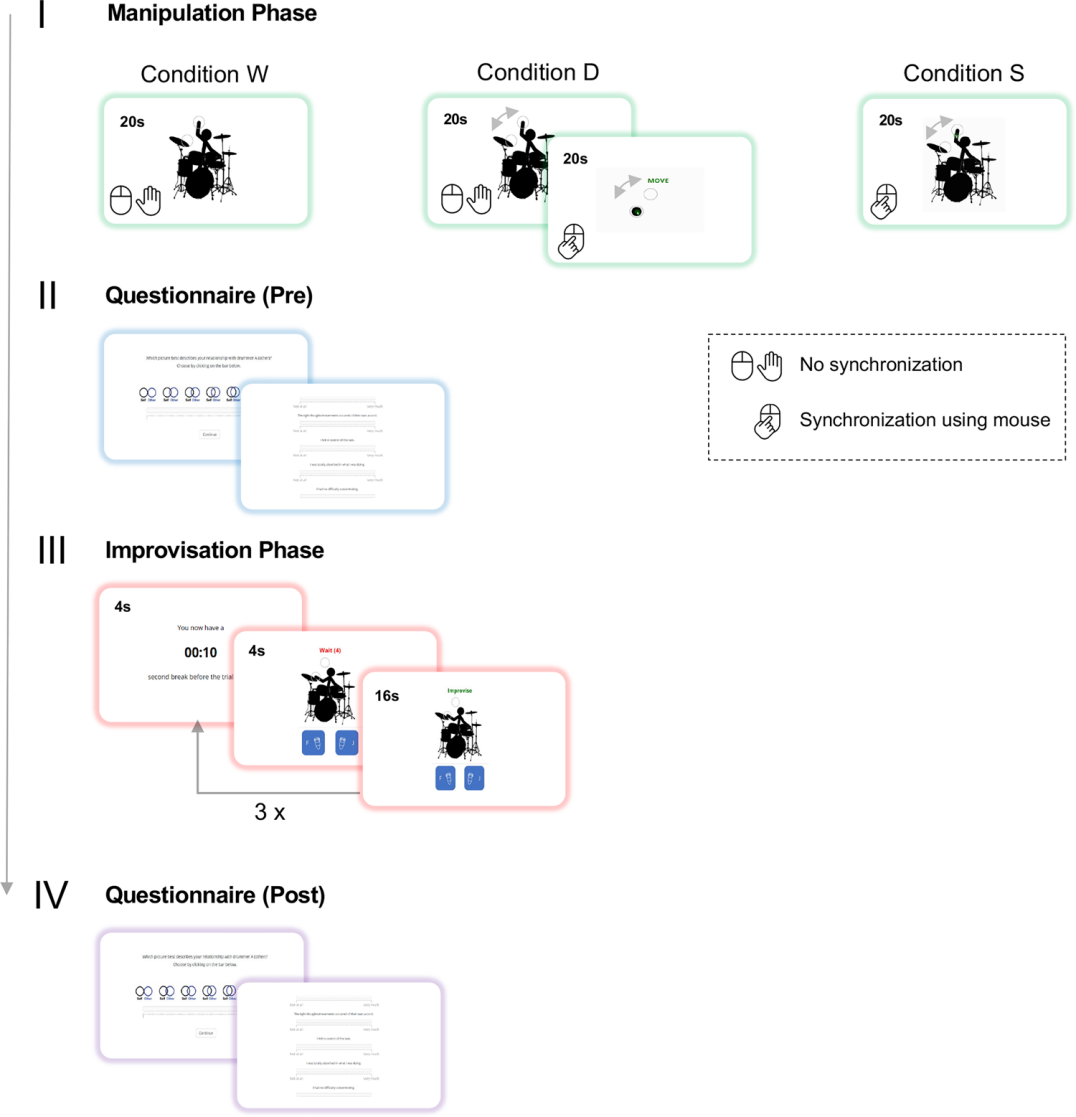
Procedure of experiment 2. Each participant ran through the procedure three times (within-subject design), each time engaging in a different condition in the manipulation phase: In condition “(W)atch”, participants passively watched the drummer play a drum beat; condition “(D)ot” involved the same task before participants were asked to synchronize with a moving dot; in condition “(S)ynchronize”, participants moved along with the drummer avatar using their mouse cursor. Subsequently, participants completed a questionnaire (pre) before they were asked to perform three short improvisations with the avatar during the improvisation phase. Participants could improvise by triggering two conga samples (blue buttons on the screen) via their keyboard while they saw and heard the avatar play a drumbeat. After latter phase participants completed a questionnaire (post) identical to the one before the improvisation phase. Participants completed all three experimental conditions a single time including the condition W (“Watch”), condition D (“Dot”) and condition S (“Synchronize”).

The experiment began with a test trial phase where participants could get accustomed to the manipulation and improvisation task. The test trial phase was followed by a practice phase in which participants could practice the improvisation task, namely triggering conga samples via their keyboard. In the practice phase, they were not accompanied by a backing track. Participants had to practice for at least one minute but could go on for up to 10 min. Afterwards, participants were asked to complete all three experimental conditions (the experimental procedure is depicted in Fig. 3). The three conditions were fully counterbalanced, and in each, the avatar was given a different acronym (A/B/C) and a different color (e.g., red). Only in the test trial phase, the avatar had no name and a neutral (black) color. The conditions always started with a manipulation phase involving one of the manipulation tasks. In every condition, the manipulation phase concluded with the same questionnaires (IOS and FSS) also used in the first study (see Supplementary Table S1). After each manipulation phase, participants took part in an improvisation phase involving three trials of the improvisation task with a 10 s pause in between. Finally, participants were invited to answer the same questionnaire administered before the improvisation phase, starting again with the IOS scale. A video recording of the experimental procedure can be accessed via the following link: https://youtu.be/p-IetbPfp24.

All the recorded improvisations were assessed by the expert musicians of the rater group. Each expert rated all 162 (18 × 9) improvisations in a randomized order using a six-item questionnaire (see Supplementary Table S2). The questionnaire assessed general facets of the improvisation as likeability and how well participants played together with the backing track. Creativity was assessed by a direct question concerning the creativity of the improvisation but also by covering aspects of creativity as appropriateness. However, we considered only the direct question on creativity in our data analysis. The questionnaire was administered in the rater’s native language, either English or German. Raters were asked to listen to the improvisation at least one time and to respond to every rating question before continuing to the next improvisation. They could listen to each improvisation as many times as they needed to, and no time limit was given.

## Results

**Figure 4:**
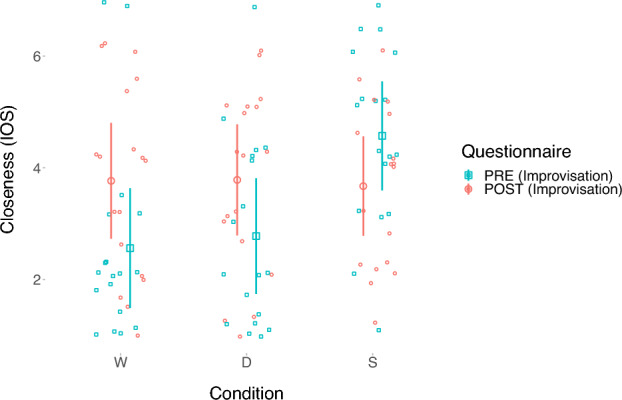
Visualization of the mean closeness (IOS) as measured before and after the improvisation phase in each condition. The error bars display 95% confidence intervals, corrected as proposed by Morey^65^. W stands for Condition “Watch”; D stands for Condition “Dot”; and S stands for Condition “Synchronize”.

We computed linear mixed models using the “lmer” function from the package “lme4”^35^ in R^36^. The “emmeans”^41^ package was used to obtain condition means and slopes from our models if necessary, and the latter package was also used to calculate post-hoc comparisons with *p*-values adjusted according to the Tukey method. All continuous variables were z-standardized using the R package “parameters”^42^. Firstly, we were interested in the dynamics of the felt closeness as measured on the IOS scale during each condition. The average IOS in each condition before the improvisation phase IOS(pre) and after the improvisation phase IOS(post) is depicted in Fig. 4. In the first analysis, we examined the direct effect of the manipulation phase on the IOS before and after the improvisation phase, that is, IOS (pre) and IOS (post), respectively. We constructed a (Model 2.1) for IOS(pre) as outcome variable with condition as predictor and a varying intercept for subject. The predictor condition was coded using treatment contrasts^37^. Thus, the model tested the differences between the conditions “Dot” and “Synchronize” and the baseline condition “Watch”. While we found no significant difference (β_1_ = 0.11, 95% CI [− 0.26, 0.49]) between condition “Dot” and condition “Watch”, we observed a significantly higher IOS (β_2_ = 1.06, 95% CI [0.68, 1.44]) in the condition “Synchronize” compared to the baseline condition “Watch”. A similar (Model 2.2) was constructed with IOS(post) as outcome variable. Post-hoc comparisons between conditions did not show any significant differences (“Watch”—“Dot”: *t*(34) = − 0.06, *p* = 0.998; “Watch”—“Synchronize”: *t*(34) = 0.34, *p* = 0.94; “Dot”—“Synchronize”: *t*(34) = 0.11, *p* = 0.918) in IOS after the improvisation phase. See Table 2 for a detailed summary of the linear models.

**Table 2:**
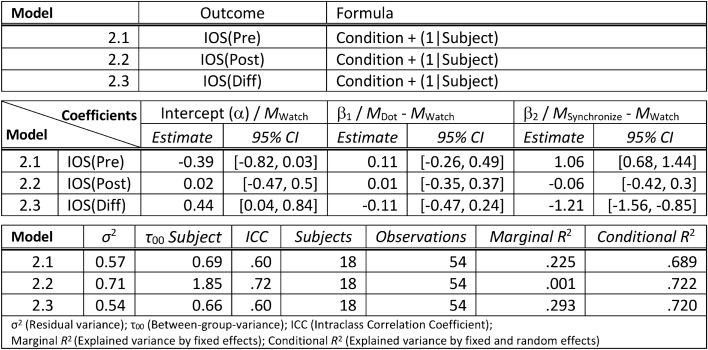
Summary of the linear mixed models (presentation similar to^66^) to test for difference in IOS(Pre) (measured before the improvisation phase), IOS(Post) (measured after the improvisation phase) and IOS(Diff) (the difference between IOS(Pre) and IOS(Post)). The models use treatment contrasts to test for: (1) the difference between the outcome variable in condition “(D)ot” and the outcome variable in the baseline condition “(W)atch” and (2) the difference between the outcome variable in condition “(S)ynchronize” and the outcome variable in the baseline condition “(W)atch”. The intercept of the models is the mean of the outcome variable in the baseline condition “(W)atch”. The model formulas are stated in the lme4 syntax.

In a second analysis we investigated the difference in IOS before and after the improvisation using a linear mixed model. We therefore built a (Model 2.3) inspecting IOS(diff) = IOS(post)—IOS(pre) as outcome variable, condition as predictor and a varying intercept for subject. Treatment contrasts were applied to the predictor condition (see Table 2 for a detailed summary of the linear model). We estimated the mean IOS(diff) for every condition using the “emmeans”^41^ package which showed that in condition “Watch” and “Dot” the IOS increased significantly (“Watch”: *M* = 1.21, 95% CI [0.49, 1.92]; “Dot”: *M* = 1.01, 95% CI [0.29, 1.72]) during the improvisation phase. In contrast, the IOS in condition “Synchronize” decreased significantly (*M* = − 0.90, 95% CI [− 1.61, − 0.18]). Indeed, post-hoc comparisons showed that the change of IOS during the improvisation did significantly differ between the condition “Synchronize” and either of the conditions “Watch” and “Dot” (“Watch”—“Synchronize”: *t*(34) = 6.74, *p* < 0.001; “Dot”—“Synchronize”: *t*(34) = 6.10, *p* < 0.001), while conditions “Watch” and “Dot” did not significantly differ (*t*(34) = 0.64, *p* = 0.80) in their change of IOS during the improvisation.

**Figure 5:**
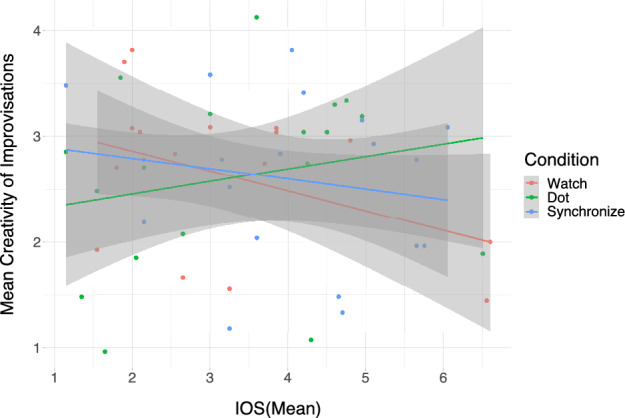
Visualization of the correlations between IOS(mean) and the mean creativity of improvisations for each condition including 95% confidence intervals. The figure also depicts the mean creativity rating of each improvisation at the IOS(mean) for each participant in each condition. There is as significant negative correlation in condition “Watch” and “Synchronize” between IOS(mean) and the improvisations’ creativity. Though, there is no significant correlation between IOS(mean) and the improvisations’ creativity in condition “Dot”.

Finally, we were interested in investigating the rated creativity of improvisations across the three conditions. On average participants’ improvisations were given a mean creativity rating of *M*_*Creativity*_ = 2.6 (*SD* = 1.57) on a 7-point Likert scale where a rating of 1 indicated that the judge “strongly disagreed” that the improvisation is creative. Furthermore, to assess the improvisational fluency of our participants, we tallied the number of notes (#Notes) played in each improvisation trial, indicating how often they triggered either conga sample. Subsequently, the number of notes (#Notes) was averaged across all conditions and participants to calculate the measurement of improvisational fluency, denoted as *M*_*#Notes*_*.* Overall, participants showed a good fluency in improvising on the virtual conga set, performing on average *M*_*#Notes*_ = 108.1 (*SD* = 40.81, Range = [42, 192]) notes during each 15 s of improvisation. We again used a linear mixed model to test for a difference in creativity between conditions. Our model also included the interaction of condition with the mean IOS of each condition, i.e. IOS(mean), which further allowed us to directly examine correlations between measured IOS and creativity in each condition. We included IOS(mean) instead of IOS(pre) or IOS(post) in our model as we noticed in previous analyses that the IOS seemed to change throughout the conditions and the mean may best reflect the average IOS during the improvisation phase. The (Model 2.4) with the outcome variable of rated creativity included condition and IOS(mean) as predictors, as well as their interaction. Additionally, the model included a varying intercept for subject and rater. These varying intercepts for the subject and rater allowed us to account for variation in rated creativity between every combination of subject and rater. Conditions were coded using treatment contrasts (relative to the passive condition “Watch”). To calculate (Model 2.4) we used the “lmer” function from the “R” package “lme4″ with the “bobyqa” optimizer. The improvisations did not differ in their rated creativity between conditions (“Dot”—“Watch”: β = 0.02, 95% CI [− 0.09, 0.13]); “Synchronize”—“Watch”: β = 0.05, 95% CI [− 0.07, 0.18]). We obtained the estimations for the correlation between the IOS(mean) and creativity in each condition using the “emmeans”^41^ package. In condition “Watch” and “Synchronize” the IOS(mean) showed a significantly negative correlation (“Watch”: *M* = − 0.15, 95% CI [− 0.27, − 0.03]; “Synchronize”: *M* = − 0.15, 95% CI [− 0.28, − 0.02]) with the improvisations’ creativity, while the correlation was not significant (”Dot”: *M* = 0.09, 95% CI [− 0.02, 0.20]) in condition “Dot”. Post-hoc comparisons showed that the correlation between creativity and IOS(mean) in condition “Dot” significantly differed (“Watch”—“Dot”: *t*(1412) = − 4.02, *p* < 0.001; “Dot”—“Synchronize”: *t*(1416) = 0.24, *p* < 0.001) from the other two conditions “Watch” and “Synchronize” and no significant difference (“Watch”—“Synchronize”: *t*(1413) = − 0.03, *p* = 1) was found between condition “Watch” and “Synchronize. (See Table 3 for a detailed summary of the model and Fig. 5 for a visualization of the correlations between IOS(mean) and the improvisations’ creativity in each condition)*.* It should be noted that our models exploring the influence of condition and measured IOS explain only little variance, as indicated by the low *R*^2^ values. This is not completely unexpected as creativity and the influence of social cohesion are highly complex topics with multiple cofounding variables that cannot be controlled or included within a simple model.

**Table 3:**
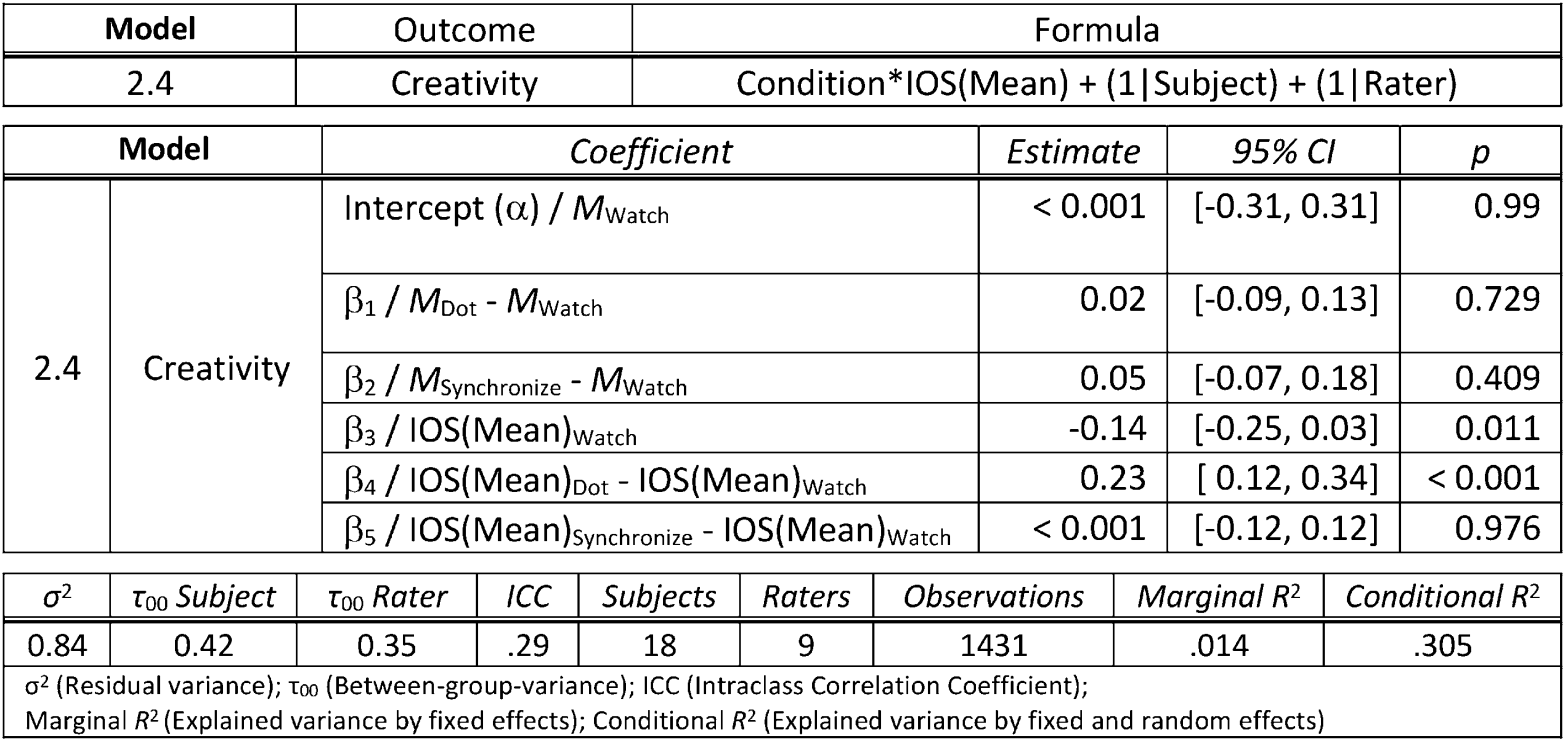
Summary of the linear mixed model (presentation similar to^66^) to test for differences in creativity ratings between conditions in interaction with the influence of the IOS(mean). The model uses treatment contrasts to test for: (1) the difference in mean creativity between the condition “(D)ot” and the baseline condition “(W)atch” and (2) the difference in mean creativity between the condition “(S)ynchronize” and the baseline condition “(W)atch”. The intercept of the model is the mean creativity in the baseline condition “(W)atch”. Furthermore, the model estimates the correlation IOS(mean)_Watch_ between IOS and creativity in condition “(W)atch”. It also assesses how much this correlation differs in conditions “(D)ot” and “(S)ynchronize”. The model formula is stated in the lme4 syntax.

## General discussion

We performed two experiments to investigate the role of social cohesion in creativity, focusing on a musical context. In the first experiment, we explored if synchronized movement with a virtual avatar drummer can increase social cohesion (as measured on the IOS scale). In the second experiment, we tested whether such changes in social cohesion influence creativity in an improvisation task together with the avatar.

Three main results were obtained: (1) In both studies we found that music-induced interpersonal synchronization with an avatar increases the feeling of social cohesion as rated on the IOS scale. (2) In the second study we additionally observed that social cohesion reached an increased level after the improvisation phase that was comparable across conditions. (3) An increased feeling of social cohesion in the second study was associated with significantly lower creativity of improvisations in conditions “Watch” and “Synchronize”, where respectively participants watched the avatar without moving, or synchronized with it. Yet, we found condition “Dot” (where participants first watched the drummer and afterwards synchronized with a moving dot) to significantly differ in respect to this correlation. Our findings stand in contrast to our proposed hypothesis in which we assumed that social cohesion is positively correlated with creativity. Against our expectations, we did not find an effect on creativity of the condition “Synchronize” when comparing it to the baseline condition “Watch”. We anticipated creativity to differ in condition “Synchronize” due to the interpersonal synchronization task and the increased level of social cohesion.

Our first main result (i.e., music-induced interpersonal synchronization with an avatar increases the feeling of social cohesion as rated on the IOS scale) is in line with previous findings showing that interpersonal synchronization increases the feeling of social cohesion in live as well as virtual settings^19,23,24^. We extend this research by showing that such an effect also arises when participants synchronize overtly with a virtual avatar. This result was obtained in our first study and was replicated in the second. The underlying cognitive mechanism why interpersonal synchronization increases the feeling of social cohesion is still a matter of debate. Among other theories, it has been suggested that the alignment of movements with others results in an alignment of mental states^31,43^. Such merging of self and the other has been shown to underly the feeling of social cohesion^32,33,44^. Moreover, the alignment of mental states makes co-actors more predictable and helps in optimizing interactions by decreasing effort and cognitive load^45^, resulting in an increased willingness to cooperate^46,47^ and hence prosocial behavior and attitude.

Our second main result (i.e., social cohesion reached an increased level after the improvisation phase that was comparable across conditions) is especially interesting as we did not expect social cohesion to reach similar levels in conditions “Watch” and “Dot” as in “Synchronize”. The former two conditions neither involved an interpersonal-synchronization task, nor did they show heightened social cohesion directly before the improvisation phase (that is, after the manipulation phase). A possible explanation could be that carrying out the improvisation task together with the avatar might have induced a sense of closeness to the virtual drummer which compensated for the absence of the interpersonal synchronization task in the conditions “Watch” and “Dot”.

Hence, the improvisation phase might have positively influenced social cohesion. However, it should be noted that closeness (as assessed through the IOS) only increased (from pre to post improvisation) in conditions “Watch” and “Dot”. In contrast, in condition “Synchronize” the level of IOS *decreased* to reach the same elevated level as in the other conditions. Considering our interpretations and that all conditions involved the same improvisation task, it might be argued that the level of social cohesion in condition “Synchronize” should have stayed constant or even increased. A possible explanation for this might be that the effect we found in condition “Synchronize” is confounded by the participants’ expectation (see Atwood and colleagues^48^): participants might have expected their social connectedness to increase by performing an interpersonal-synchronization task with the avatar. Hence, this effect observed directly after the manipulation phase might only partially reflect an implicit measurement of social cohesion. In other words, we suggest that the increase on the IOS scale before the improvisation phase in condition “Synchronize” might have been caused by the participants’ expectation of such an effect. Accordingly, the levels of IOS before and after the improvisation phase in condition “Synchronize” may not be comparable to the other conditions. Hence, we should not advance any conclusions based on the seemingly contradictory dynamics of the IOS in condition “Synchronize”, when compared to the other conditions. Further research is needed to precisely determine why the IOS increased during the improvisation phase in condition “Watch” and “Dot”.

We note that our third result (i.e., an increase in social cohesion is associated with lower rated creativity of improvisation in conditions “Watch” and “Synchronize”, but not in condition “Dot”), stands in contrast to studies where creativity is seen to benefit from social cohesion^8–10^. Instead, our finding resonates with research that shows how social cohesion and interpersonal synchrony can impede creativity^49,50^. Yet, while condition “Dot” did not differ in terms of measured social cohesion from condition “Watch”, we did find the correlation between social cohesion and creativity to be dissimilar in condition “Dot”.

The divergence between the association of social cohesion and creativity in condition “Watch” and “Synchronize” compared with condition “Dot” could be explained in terms of differences in self-construal. This pertains to how independent or interdependent individuals define themselves in relation to others, in this context with the avatar^51^. Research has pointed out that self-construal is influenced by factors as social cohesion in their effect on group creativity^52–54^. The experimental procedure in condition “Watch” and “Synchronize” permanently guides participants’ attention towards the avatar and the collective task of improvising. In both conditions participants engage with the virtual drummer throughout the whole condition by either watching, synchronizing, or improvising with the avatar. Thereby, we could have induced more interdependent self-construal in participants. Hence, during these two conditions they perceive their task efforts and goals completely in relation to the avatar. In comparison, the procedure in condition “Dot” features a task which participants must carry out on their own without the avatar. This might have led to more independent self-construal. Such independent self-construal within a collective task has been shown to increase creativity^53,54^. Hence, in condition “Dot” it might have mitigated the negative effect of social cohesion on creativity that we found in condition “Watch” and “Synchronize”. In the latter conditions creative performance might have been impeded by the combination of social cohesion and interdependent self-construal leading to excess conformity^55^. Further investigations are needed to deepen our understanding of this potential interaction between social cohesion and self-construal in creative group tasks such as musical improvisation. Individuals might only be able to capitalize on the social cohesion to their group members if they sufficiently differentiate themselves, as shown by Bechtoldt and colleagues^53^. Similar observations have been made in research on creativity in free musical improvisation. Synchronization, used for coordination and facilitating social cohesion, is a prerequisite of successful improvisation^56,57^. Nevertheless, creativity seems to especially profit from moments of dissensus through non-interaction and non-cooperation during the coordinated joint-activity^58,59^. However, independent self-construal might be necessary as a first step to allow diverging from the collective group behavior.

Before we conclude, we would like to discuss possible limitations of our work. Firstly, the findings of our second study should be interpreted with caution due to the low sample size of 18 participants. As we have pointed out, a diligent assessment of all improvisations by expert judges is very time consuming and limited the number of participants in our study. However, we think that our paradigm is promising and a meta-analysis of further studies using a comparable paradigm will allow to generalize our findings further. Secondly, the present research focuses solely on the improvisation of rhythm while musical improvisation often features variation in rhythm and pitch. However, we want to point out that this approach allowed us to conduct the study in a general population without musical training, as there was no need for accurate pitch production or knowledge about musical harmony. Yet, future work is necessary to develop musical creativity tasks which allow participants independent of musical expertise to express themselves also using pitch and harmony. Only by studying each of these aspects in isolation as well as in combination we will gain an in-depth understanding of the creative processes involved in making music. However, we argue that the focus on rhythm does not necessarily restrict the generalizability of our study as rhythmic improvisation shares many neural correlates with melodic improvisation^17^. We argue that concentrating on rhythmic improvisation might even be particularly suited to study musical creativity in context of the research on domain-general creativity. It has been shown that rhythmic skills are not domain-specific to music but for example also determine linguistic abilities^60^. Hence, the relationship between creativity in musical improvisation and domain-general assessments^61,62^ might be particularly pronounced in rhythmic improvisation tasks. Secondly, our contribution is limited in that the virtual partner is non-adaptive and consists solely of a simple animation created from two static images. Hence, the virtual partner does not perform ecologically valid movements on the drum set. This could create the impression that the virtual partner is not actively playing the drumbeat or that its movements are slightly misaligned with the drumbeat. Yet, our participants have not reported any concerns in this regard. This is likely because they lack musical training and consequently have little expectations regarding the exact movements required to produce a drumbeat. Nevertheless, further research with more realistic and adaptive partners or real human partners (see e.g., Fairhurst and colleagues^63^ and Washburn and colleagues^64^) is necessary to extend our results. Using more realistic improvisation partners might also allow us to include improvisers with different levels of expertise, thereby overcoming the restriction to people with no musical training in our current study.

To conclude, our study provides further evidence showing that overt synchronization with a virtual avatar increases social cohesion. Moreover, we have explored the effect of social cohesion on creativity in a task where participants improvised musical rhythms. Our findings suggest that increased social cohesion might be associated with less creative outcomes in improvisation. However, as discussed, the exact circumstances under which social cohesion leads to less creative outcomes need to be further studied. Novel research is necessary to help generalize our findings and examine whether these replicate using other musical improvisation tasks apart from rhythmic production (e.g., featuring also melody and harmony) as well as in domains beyond music. That said, rhythmic improvisation might still be a well-suited task to study the interplay of social contingencies in group creativity independent of domain-specific expertise, as it offers an ecological approach through the dynamics of synchronization and social cohesion.

### Supplementary Information

Supplementary Tables.

## Data Availability

Data and code used in this study is available at https://osf.io/pcmdt/?view_only=1e6bdac31cb64f8c8677a50b9523edcc.
