# Supplementary material for: An observation of a negative effect of social cohesion on creativity in musical improvisation
Source: Sci Rep. 2024 Feb 5;14:2922. doi: 10.1038/s41598-024-52350-7 (PMC10844246; doi:10.1038/s41598-024-52350-7)
Supplement: Supplementary file 1 — Supplementary Tables. [file 41598_2024_52350_MOESM1_ESM.docx]

**An observation of a negative effect of social cohesion on creativity in musical improvisation.**

Adrian Kempf [1] *, Mathias Benedek [2], Andrea Schiavio [3, 1]

[1] Department of Psychology, University of Graz, Glacisstraße 27, Graz, 8010, Austria.

[2] Department of Psychology, University of Graz, Graz, Austria.

[3] School of Arts and Creative Technologies, University of York, UK.

*Corresponding author. E-mail: [adrian.kempf@uni-graz.at](mailto:adrian.kempf@uni-graz.at)
Contributing authors: [adrian.kempf@](mailto:adrian.kempf@)uni-graz.at; [mathias.benedek@uni-graz.at](mailto:mathias.benedek@uni-graz.at); [andrea.schiavio@york.ac.uk](mailto:andrea.schiavio@york.ac.uk)

**Supplementary Information**

**Table S1**: Questions used in experiment 1 and 2.

| ID | Question | Type | Range | Granularity |
| --- | --- | --- | --- | --- |
| A | Which picture best describes your relationship with drummer A/B/C (other)? Choose by clicking on the bar below. | Continuous | [1, 7] | 0.1 |
| B | How much did you like drummer A/B/C (D/E/F) ? | Continuous | [1/Not at all, 10/Very much] | 0.1 |
| C | How synchronized did you feel with drummer A/B/C (D/E/F)? | Continuous | [1/Not at all, 10/Very much] | 0.1 |
| D | How much did you like the drum beat? | Continuous | [1/Not at all, 10/Very much] | 0.1 |
| E | I felt just the right amount of challenge during the task. | Continuous | [1/Not at all, 10/Very much] | 0.1 |
| F | My thoughts/activities were running fluidly and smoothly. | Continuous | [1/Not at all, 10/Very much] | 0.1 |
| G | I didn't notice time passing. | Continuous | [1/Not at all, 10/Very much] | 0.1 |
| H | I had no difficulty concentrating. | Continuous | [1/Not at all, 10/Very much] | 0.1 |
| I | My mind was completely clear. | Continuous | [1/Not at all, 10/Very much] | 0.1 |
| J | I was totally absorbed in what I was doing. | Continuous | [1/Not at all, 10/Very much] | 0.1 |
| K | The right thoughts/movements occurred of their own accord. | Continuous | [1/Not at all, 10/Very much] | 0.1 |
| L | I knew what I have to do each step of the way. | Continuous | [1/Not at all, 10/Very much] | 0.1 |
| M | I felt in control of the task. | Continuous | [1/Not at all, 10/Very much] | 0.1 |
| N | I was completely lost in thought. | Continuous | [1/Not at all, 10/Very much] | 0.1 |
| O | At the moment I am in a good mood. | Continuous | [1/Not at all, 10/Very much] | 0.1 |
| P | For me personally, the current demands were ... | Continuous | [1/Too low, 5/Just Right 10/Too high] | 0.1 |
| Q | How much did you move your body during listening to drummer A/B/C (D/E/F)? | Continuous | [1/Not at all, 10/Very much] | 0.1 |

**Table S2**: Evaluation questions for expert raters on which every improvisation was assessed.

| ID | Question | Type | Range | ICC | 95 % CI |
| --- | --- | --- | --- | --- | --- |
| A | In my opinion, the improviser plays well together with the drummer. / Ich finde, dass der Improvisator gut mit dem Schlagzeuger zusammenspielt. | Discrete | [1/Strongly disagree, 7/Strongly agree] / [1/Trifft überhaupt nicht zu, 7/Trifft völlig zu] | 0.78 | [0.72594, 0.8278068] |
| B | In my opinion, the improvisation is creative. / Ich erachte die Improvisation als kreativ. | Discrete | [1/Strongly disagree, 7/Strongly agree] / [1/Trifft überhaupt nicht zu, 7/Trifft völlig zu] | 0.79 | [0.737017, 0.8347666] |
| C | In my opinion, the improvisation is unusual. / Ich erachte die Improvisation als ungewöhnlich. | Discrete | [1/Strongly disagree, 7/Strongly agree] / [1/Trifft überhaupt nicht zu, 7/Trifft völlig zu] | 0.70 | [0.6266374, 0.7654145] |
| D | In my opinion, the improvisation is appropriate. / Ich erachte die Improvisation als angemessen. | Discrete | [1/Strongly disagree, 7/Strongly agree] / [1/Trifft überhaupt nicht zu, 7/Trifft völlig zu] | 0.41 | [0.2620453, 0.5363396] |
| E | I like the improvisation. / Mir gefällt die Improvisation. | Discrete | [1/Strongly disagree, 7/Strongly agree] / [1/Trifft überhaupt nicht zu, 7/Trifft völlig zu] | 0.71 | [0.6330916, 0.7694697] |
| F | I was surprised by the improvisation. / Mich hat die Improvisation überrascht. | Discrete | [1/Strongly disagree, 7/Strongly agree] / [1/Trifft überhaupt nicht zu, 7/Trifft völlig zu] | 0.72 | [0.6517897, 0.7812178] |
